# Adjuvant therapy with minocycline for schizophrenia (The MINOS Trial): study protocol for a double-blind randomized placebo-controlled trial

**DOI:** 10.1186/1745-6215-14-406

**Published:** 2013-11-27

**Authors:** Abebaw Fekadu, Miraf Mesfin, Girmay Medhin, Atalay Alem, Solomon Teferra, Tsehaysina Gebre-Eyesus, Teshale Seboxa, Abraham Assefa, Jemal Hussein, Martha T Lemma, Christina Borba, David C Henderson, Charlotte Hanlon, Teshome Shibre

**Affiliations:** 1 Department of psychiatry, School of Medicine, College of Health Sciences, Addis Ababa University, Addis Ababa, Ethiopia; 2 King’s College London, Institute of Psychiatry, Department of Psychological Medicine, Affective Disorders Research Group and Centre for Affective Disorders, London, UK; 3 Aklilu Lemma Institute of Pathobiology, Addis Ababa University, Addis Ababa, Ethiopia; 4 Department of Internal Medicine, St Paul Hospital Millennium Medical College, Addis Ababa, Ethiopia; 5 Department of Internal Medicine, Section of infectious diseases, College of Health Sciences, Addis Ababa University, Addis Ababa, Ethiopia; 6 Armauer Hansen Research Institute, ALERT, Addis Ababa, Ethiopia; 7 Department of Microbiology, Bahirdar University, Bahirdar, Ethiopia; 8 The Chester M. Price, MD Division of Global Psychiatry, Massachusetts General Hospital, Harvard Medical School, MGH Schizophrenia Program, Boston, MA, USA; 9 Department of Health Services and Population Research, King’s College London, Institute of Psychiatry, London, UK

**Keywords:** Minocycline, Schizophrenia, Intervention, Clinical trial, Ethiopia

## Abstract

**Background:**

Schizophrenia is understood to be a heterogeneous brain condition with overlapping symptom dimensions. The negative symptom dimension, with its protean cognitive manifestations, responds poorly to treatment, which can be a particular challenge in countries where clozapine therapy is not available. Preliminary data indicate that minocycline may be beneficial adjunct in the treatment of schizophrenia: positive, negative, and cognitive symptoms.

In this study we aim to assess the efficacy of adjunctive minocycline to alleviate symptoms of schizophrenia in patients who have failed to respond to a therapeutic trial of antipsychotic medications.

**Methods:**

The study is a parallel group, double-blind, randomized, placebo-controlled trial. Participants will be adults (aged 18 years and above) with first episode or relapse episode of schizophrenia of under 5 years’ duration. Patients who failed to show adequate therapeutic response to at least one antipsychotic medication given for a minimum of 4 weeks will be recruited from a psychiatry hospital in Addis Ababa and a psychiatry clinic in Butajira, Ethiopia. A total of 150 participants (75 in each arm) will be required to detect a five-point mean difference between the intervention arms adjusting for baseline symptom severity, at 90% power and 95% confidence. Patients in the intervention arm will receive minocycline (200 mg/day orally) added on to the regular antipsychotic medications participants are already on. Those in the placebo arm will receive an inactive compound identical in physical appearance to minocycline. Intervention will be offered for 12 weeks. Diagnosis will be established using the operational criteria for research (OPCRIT). Primary outcome measure will be a change in symptom severity measured using the positive and the negative syndrome scale for schizophrenia (PANSS). Secondary outcome measures will include changes in severity of negative symptoms, proportion achieving remission, and level of functioning. Whether changes are maintained post intervention will also be measured (PANSS). Key assessment for the primary outcome will be conducted at the end of trial (week 12). One post-intervention assessment will be conducted 4 weeks after the end of intervention (week 16) to determine sustainability of change.

**Trial registration:**

Clinicaltrials.gov identifier: NCT01809158.

## Background

Schizophrenia is a severe mental disorder associated with substantial morbidity and burden. For many individuals it causes a lifelong disability at immense cost to family and society [1]. Schizophrenia is accepted to be a heterogeneous brain disorder with undetermined etiology. The conceptualization of schizophrenia has evolved over the past century, since its distinction from affective psychosis. The diagnosis rests on the combination of the occurrence of typical clinical features and longitudinal observation. Based on the clinical features, three overlapping dimensions of schizophrenia are identified: positive, negative, and disorganized [2,3]. These dimensions have a significant bearing on the course of the disorder: those with negative symptoms have poorer treatment response and more unfavorable course. Negative symptoms are particularly associated with various cognitive impairments that may lead to poor treatment response.

Although the introduction of antipsychotic medication almost half a century ago was undoubtedly a huge step forward in the treatment of schizophrenia [4], a large proportion of patients fail to respond satisfactorily to trials with antipsychotic medications. For example, a study of 1,400 people with schizophrenia from 52 diverse sites across the United States found that only 14.5% participate in gainful employment, 12.9% report work in workshops or rehabilitation programs, and 72.6% reported no employment [5]. In the past 15 years, several new medications called second generation or atypical antipsychotics have been introduced for the treatment of schizophrenia. These new generation antipsychotic medications have shown substantially fewer extrapyramidal side effects (EPSE) than first generation antipsychotic medications, which are the mainstay of treatment in low income countries like Ethiopia. However, these second generation antipsychotics are not shown to be clinically more effective than first generation antipsychotics [6] and are associated with metabolic side effects. The only second generation antipsychotic medication which has been shown to be clinically more effective and to reduce rates of hospitalization is clozapine [7,8]. Moreover, the side effect burden of clozapine is high and clozapine is not available in many low income countries, including Ethiopia.

Preliminary data indicate that minocycline, a safe semi-synthetic tetracycline, may have neuroprotective properties and efficacy in improving negative symptoms of schizophrenia. Minocycline may be particularly relevant in low income countries, where it has been hypothesized that infectious agents contributing to the presentation of schizophrenia may be more common. In this study we aim to assess the efficacy of minocycline to alleviate symptoms of schizophrenia when given as an adjuvant to ongoing antipsychotic treatment.

### Minocycline: nature

Minocycline is a tetracycline compound with activity as a broad spectrum bacteriostatic antibiotic. Minocycline is intrinsically more active against gram-positive than gram-negativemicro-organisms. It is also effective against micro-organisms such as rickettisia, coxiellaburnetti, mycoplasmapneuomniae, chlamydia species, legionella species, ureaplasma, some atypical mycobacteria, and plasmodium species. It is also active against many spirochetes, including borrelia burgdorferi (lyme disease) and treponema pallidum (syphilis). Minocycline inhibits bacterial protein synthesis by binding to the 30s bacteria ribosome and preventing access of aminoacyl tRNA to the acceptor (A) site on the mRNA-ribosome complex [9].

After an oral dosing, mincycline is almost completely absorbed and has a half-life of 16 to 18 h. It can therefore be administered less frequently and at lower doses. Importantly, unlike many other tetracyclines, food (including dairy products) does not interfere with the absorption of minocycline. It is significantly metabolized by the liver and is recovered from the urine and feces in lower amounts than other tetracyclines. The t_1/2_ of minocycline is not prolonged in patients with hepatic failure. The usual dose of minocycline is 100 mg every 12 h.

Minocycline is a well tolerated medication although some of the general side effects related to tetracyclines may occur. Gastro-intestinal irritation may occur but improves when administered with food. Hepatic toxicity typically develops in patients receiving large quantities orally. Minocycline may aggravate azotemia in patients with renal disease. Brown discoloration of teeth may also occur if given between the ages of 2 months and 5 years. Treatment with tetracycline during pregnancy may also be associated with tooth discoloration in the child. Patients taking minocycline may also experience some vestibular toxicity, manifested by dizziness, ataxia, nausea, and vomiting. Symptoms occur soon after the initial dose and usually disappear within 1 to 2 days after drug cessation. Hypersensitivity rarely occurs.

### Minocycline for neurodegenerative diseases and schizophrenia

Minocycline has excellent penetration of the blood–brain barrier and is believed to modulate inflammatory processes linked to the pathophysiological mechanisms relevant to the onset or progression of neurodegenerative diseases [10] and schizophrenia [11].

Reviewing the potential benefits of minocycline in mental disorders and schizophrenia, four pathways, which were linked to the causation of mental disorders, have been proposed [11]. The first mechanism is the calcium mediated glutamate excitotoxicity. Increased glutamate modulated intracellular calcium has been linked with mood and psychotic disorders, and minocycline was reported to reduce glutamate induced neurotoxicity in animal models. In one study, minocycline attenuated the behavioral changes following the administration of an NMDA antagonist in mice [12]. In another study, minocycline reversed the effects of an NMDA antagonist in rats [13]. The second mechanism highlighted relates to the antioxidant properties of minocycline. Minocylcine directly scavenges free radicals and inhibits molecules such as cyclooxygenase 2, induced nitric oxide synthase, and nicotinamide adenine dinucleotide phosphate oxidase. The third mechanism refers to the nuroprotective properties of minocycline, for example, through the caspase-independent anti-apoptotic effects including upregulation of antiapoptotic factor BCL-2 [14]. The fourth pathway relates to the anti-inflammatory properties of minocycline. In a mechanism that seems to be distinct from its antimicrobial properties, minocycline has anti-inflammatory and neuroprotective properties [15].

Even though the role of microgilia in the neruo-inlammatory process is not fully confirmed [16], the anti-inflammatory activity of minocycline is attributed partly to its capacity to suppresses neuroimmune activation/proliferation of microglia as well as subsequent release of proinflammatory cytokines such as interleukins, IL-1β and IL-6, and tumor necrosis factor (TNF-α), neurotoxic substances such as nitric oxide, ROS, and chemokines [14]. Furthermore, the cyoprotective activity of minocycline other inflammatory mediators such as the blockade of enzymatic activity of mitogen activated protein kinase 38 (P38 MAPK) which is thought to mediate inflammatory responses and cell death [17,18]. Minocycline is also linked with the inhibition of caspase-1 and caspase-3, which are involved in the generation of interleukin-1 and apoptosis, respectively [19]. Minocycline is reported to suppress the release of lipids and the activity of matrix metalloproteases (MMPs), which disrupt the blood–brain barrier [19]. Minocycline was also found to reduce prostaglandin-E2 (PGE2) formation and inhibit cyclo-oxygenase-2 (COX-2) expression in murine microglia and prostaglandin F2a (PGF2a) and PGE2 production induced by LPS in primary rat microglial cells [11]. Minocycline has also been reported to have antiviral effects against HIV and antiprotozoal effects against *Toxoplasmagondii*.

Its use in individuals with schizophrenia has been encouraged by its effects in rodent models of this disorder. Some preliminary data also suggest that minocycline may be useful in patients with schizophrenia. Two case report series have been published, one including two patients with schizophrenia [20] and the other including three patients with recent-onset acute paranoid schizophrenia [21]. An open label study of 22 patients with treatment-resistant schizophrenia, using minocycline 150 mg/d for 4 weeks, reported an improvement in both positive and negative symptoms [15,22]. Two double-blind trials have been carried out. In one study, 73 patients with schizophrenia of <5 years’ duration were randomized to minocycline 200 mg or placebo for 12 months: ‘all symptom measures improved significantly’ especially in the negative symptoms [23]. In the other study, 54 ‘early phase’ (symptoms of <5 years) patients were randomized to minocycline 200 mg/d or placebo for 6 months; the authors reported a significant improvement in negative symptoms measured using Scale for the Assessment of Negative Symptoms (SANS) and Clinical Global Impression (CGI) and a significant improvement in some test of executive function [24].

These initial findings encourage further replication. First, these studies are of comparatively small size. Second, in countries like Ethiopia where options for the treatment of schizophrenia are limited, identifying a safe alternative to clozapine is important. Related to this, most patients have limited exposure to psychotropic medications. Third, exposure to alcohol and substances is generally believed to be lower in the Ethiopian setting. Finally, given our hypothesis that infectious agents as a cause of schizophrenia may be more important in low income countries such as Ethiopia, minocycline may prove more useful. In this regard, the trial will help to explore the etiology of schizophrenia.

### Study aim and objectives

The general aim of the study is to evaluate the efficacy and tolerability of minocycline as an adjuvant medication for patients with schizophrenia, who have failed to respond to an adequate trial of an antipsychotic medication defined as a dose equivalent to chlorpromazine of at least 200 mg per day given for a minimum of 4 weeks.

The specific objectives of the study are to determine the efficacy of minocycline added onto a standard treatment of schizophrenia in improving symptoms of schizophrenia (as measured by PANSS), global clinical state, negative symptoms of schizophrenia, and general functioning; and to assess the side effect burden of minocycline above and beyond standard antipsychotic medications.

### Hypothesis

Persons with early phase or recently relapsed schizophrenia who are prescribed minocycline in addition to standard antipsychotic medication will show greater symptom reduction (measured with the PANSS) compared with those taking standard antipsychotic medication alone.

## Methods

### Design

The study will be a double-blind, randomized, placebo-controlled study of the efficacy of adjuvant minocycline in reducing the severity of illness in treatment-resistant schizophrenia, compared with a placebo. The study will be powered to detect a minimum of five-point difference in PANSS or a minimum effect size of 0.31.

### Study setting and participants

The trial will recruit patients from the inpatient and outpatient units of Amanuel specialist psychiatric hospital in Addis Ababa and a general hospital in Butajira, southern Ethiopia. Amanuel hospital is the main institution for the care of those with severe mental illness. The hospital runs both inpatient and outpatient services. At the outpatient service over 150,000 people with mental disorder, most with severe mental illness, are seen every year. The inpatient unit has around 280 beds. Analysis of data from the inpatient unit indicates that most patients were admitted with schizophrenia [25]. Recent changes in the service structure of the hospital acknowledge this. Thus most of the clinical service is geared towards providing care for patients with schizophrenia. The hospital also serves as a repository of most psychotropic medications. For example, the Special Pharmacy holds various medications, including atypical antipsychotics (risperidone and olanzapine) for those able to pay for their medication.

Butajira hosts a large, population-based cohort study looking at the outcome of severe mental disorders (schizophrenia, bipolar disorder, and major depression) [26-28]. The study, funded by the SMRI, has been running for just over 10 years. Most included in the cohort are people with schizophrenia. Over 200 individuals with schizophrenia are currently being followed up. This is a well characterized cohort, with detailed clinical, functional, and socio-demographic information.

### Participants

**Figure 1:**
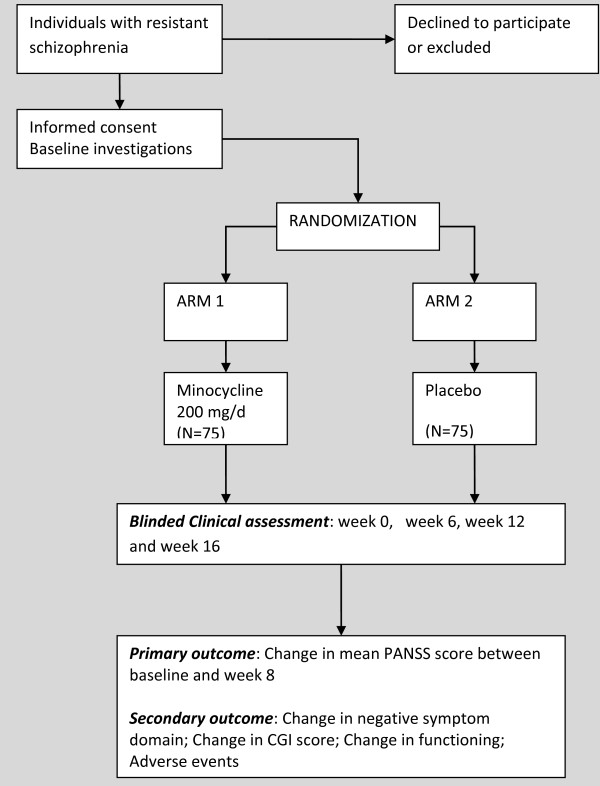
**Adjunctive minocycline *versus *** **placebo: a trial flow diagram.**

To be eligible, potential participants have to be either part of the Butajira study mentioned above or reside within the study catchment area in Butajira. If recruited from Amanuel hospital, they should be from Addis Ababa. This is so that successful follow-up can be maximized. Specifically, consenting individuals fulfilling entry criteria described below will be successively recruited. Figure 1 depicts the overall recruitment procedure.

#### Participant inclusion criteria

1. Age 18 to 64 years

2. Primary Axis I diagnosis (DSM-IV) [29] of schizophrenia, any subtype

3. The current episode should be either a relapse episode or a first onset schizophrenia and the duration of the relapse episode and first onset schizophrenia should be <5 years

4. Indication of treatment-resistance defined by failure of at least one adequate dose of antipsychotic medication (equivalent to chlorpromazine of 200 mg/day or more) given for at least 4 weeks. For the minority of patients receiving second generation antipsychotic medications, adequate doses will be Risperidone 4 mg/day, or Olanzapine 10 mg/day

5. Presence of at least moderate severity of illness measured according to the PANSS (score of at least 75 [30])

6. On adequate dose of antipsychotic medication at point of recruitment

7. Both genders, but women have to be of non-child-bearing age because of potential risks to pregnant women, and the difficulty of ensuring contraception

#### Participant exclusion criteria

1. Substance abuse co-morbidity or history of substance abuse/dependence within the previous 3 months

2. Impaired cognitive capacity because of a degenerative brain condition or trauma or diagnosis of mental retardation

3. Any serious medical condition that affects brain or cognitive function (for example, epilepsy, serious head injury, brain tumor, or other neurological and neurodegenerative conditions)

4. Any clinically significant or unstable medical disorder as determined by the trial physician that would preclude study participation, including congestive heart failure, abnormal liver function or disease, renal impairment. Also patients with leucopenia, anemia, and thrombocytopenia will be excluded

5. History of hypersensitivity to tetracycline

6. Patients on anticoagulant therapy

7. Patients requiring ergot alkaloids

8. Patients taking antacids containing aluminum, calcium, or magnesium, and products containing iron

9. Women of childbearing age (age 18 to 49 years)

10. Increased risk of suicide

#### Sample size

We used the Stata statistical program, version 11 (StataCorp 2009) to compute the required sample size. From a previous study [31], we estimated the average means (SD) of active intervention and placebo group to be 79 (16.3). Furthermore, we assumed that a five-point difference between active intervention and placebo will be clinically meaningful. This is based on a previous study, which found a statistically and clinically significant difference of five points on the PANSS comparing clozapine and olanzapine [8]. With a correlation coefficient of 0.7 and a mean change of five points on PANSS using Analysis of Co-variance (ANCOVA) method at three measurement points, 90% power and 95% confidence, a total of 118 participants would be required. Adjusting for 20% loss, a total of 147 participants will be needed. We therefore propose to recruit 150 participants (75 per arm).

### Randomization procedure and treatment allocation

Randomization will be blinded to the treating clinician, participating patients, and outcome assessors. Tablets for each patient will be packed in advance. A computerized system will be used to generate a randomization list. The randomization is to be organized so that, among each block of four individuals recruited to the study, there will be two allocations to the minocycline arm and two to the placebo arm. From the randomization list, labeled and sealed envelopes will be prepared containing cards with treatment allocation. The applicable set of sealed envelopes will be handed over to the designated senior pharmacist at Amanuel hospital or Butajira hospital for participants recruited at Amanuel and Butajira, respectively. These are to be kept in a secure place. Only the pharmacist is permitted to open the envelopes. Treatment allocation will be the responsibility of the designated senior pharmacist.

### Treatment schedules

All participants will continue their antipsychotic medication, which may be adjusted as required during the follow-up monitoring. The active intervention, minocycline, will be given at a dose of 100 mg 12-hourly. The comparison group will receive an inactive placebo compound of identical color, texture, and taste. The treatments will be given for 12 weeks, which is within the duration range of effective preliminary trials of minocycline adjunctive therapies [15,21-23]. The usual treatment will continue under the treating physician. Summary of the recruitment and follow-up is depicted in the trial flow diagram (Figure 1).

Trial medications will be dispensed on a 4-weekly basis by the senior pharmacist. Treatment adherence will be assessed through patient report and pill count. To assist with this, participants will be requested to come back with all empty packages and left over medications when coming for their follow-ups that coincide with medication resupply.

### Evaluation of treatment efficacy

The main assessments, as shown in Table 1, will be conducted at four time points: at baseline (before the initiation of trial medication), week 6, at the end of intervention (week 12), and 4 weeks post intervention (week 16) to evaluate whether changes have been maintained.

**Table 1:** Summary of assessment schedule and rating scales to be used

**Rating scales**	**Assessment schedule**							
	Baseline	Wk 2	Wk 4	Wk 6	Wk 8	Wk 10	Wk 12	Wk 16
Height	√							
Weight	√						√	
FBC	√			√			√	
LFT	√			√			√	
RFT	√			√			√	
OPCRIT	√							
PANSS	√			√			√	√
GAF	√			√			√	√
CGI	√			√			√	√
AER	√	√	√	√	√	√	√	√

AER = Adverse events rating; CGI = Clinical global impression; FBC = Full blood count; LFT = Liver function test; GAF = Global assessment of functioning; OPCRIT = Operational criteria for research; PANSS = Positive and negative syndrome scale; RFT = Renal function test.

### Assessments

#### Baseline assessments

##### Diagnostic assessment

We will use the Operational Criteria for Research (OPCRIT) [32] to make diagnosis of schizophrenia. The OPCRIT is an operational criteria checklist for psychotic disorders and is designed to be used with a computer program. The instrument uses some of the rating styles of the Schedules for Clinical Assessment in Neuropsychiatry (SCAN) [33] but is briefer and simpler to administer. It has established reliability and allows application of multiple diagnostic criteria [32]. The OPCRIT also allows diagnosis of co-morbid substance abuse and personality disorder. It has a checklist of symptoms, which can either be printed out (a paper and pencil version) or a computerized version, which together form the OPCRIT system. The paper version will be administered in a face-to-face interview and data will then be transferred into the computer program. The checklist is linked with extensive definitions that standardize each item to be rated. The SCAN has been in use in Ethiopia over many years administered by residents in psychiatry and psychiatrists [27,28]. TS and AF have trained in the application of the SCAN and have extensive experience in administering the SCAN. AF has experience of using the OPCRIT and has also provided training in the use of the tool. The OPCRIT will be administered for participants from Addis Ababa. For participants from Butajira, OPCRIT will be administered only for those who are not part of the ongoing cohort study described above. Participants from the ongoing cohort have extensive clinical data which includes SCAN diagnosis and additional diagnostic assessment is not necessary. Selected socio-demographic and clinical data will also be collected.

##### Physical assessment

A thorough physical (and neurological) assessment will be conducted by qualified physicians. We will also conduct liver function test, renal function test, full blood count, and investigations that may be relevant to the individual participant. The main objective of this assessment is to determine factors that may preclude use of minocycline.

##### Symptom severity assessment

PANSS [34-36] will be used as the main assessment instrument. PANSS is a 30-item semi-structured instrument widely used in the research of schizophrenia. Each item is rated on a seven-point severity continuum (1 to 7) and is completed using all available information: interview of participant, information from family, and clinical records. The scores provide several separate clinical subdomains but three subscales have been found more consistently: the positive syndrome subscale, the negative syndrome subscale, and the general psychopathology subscale [37]. These subscales are said to be normally distributed and independent of each other; they were also found to be ‘robust to the effects of mood, chronicity, medication side effects and cognition’ [37]. PANSS is also ‘sensitive and specific’ to the effects of medication in both the positive and negative symptoms of schizophrenia. The other advantage of the PANSS is the consistency in rating patients over time and illness course [37]. It has been successfully used in a trial in Ethiopia [31].

We will also use the CGI as a summary assessment of the clinical state of the participant. The CGI is one of the most widely used assessment tools to determine overall illness severity and efficacy of intervention. The instrument has three items which assess illness severity. The scale is completed entirely based on clinical judgment [37]. The current severity of illness is rated on a seven-point score ranging from 1 (when the patient is assessed to have no illness) to 7 (when the patient is among the most severely ill). CGI also allows rating of global improvement whether the improvement is caused by medication or not. Again improvement is rated on a single seven-point item, ranging from 1 (very much improved) to 7 (very much worse). The final item of the CGI helps rate overall efficacy of treatment as a factor of the overall burden of side effect of medication. When administered repeatedly it can evaluate treatment response [38] and has been employed in numerous intervention studies in schizophrenia. The CGI is sensitive to change and its ‘brevity, utility and appeal to clinical commonsense’ [37] makes it an attractive rating scale.

##### Functioning

We will administer the Global Assessment of Functioning (GAF) [29], a simple 100-point scale that assesses both functioning and general psychopathology. The scale is divided into 10-point intervals and the assessor assigns a numeric value to the functioning status of the participant.

##### Cognitive function

The cognitive items of the PANSS will be used to assess the impact of intervention on cognitive function.

#### Follow-up assessment

The PANSS will be the main outcome measure. We will also use the CGI and GAF. In addition to the key assessment dates for primary outcome (baseline, week 6, and week 12), participants will be assessed every 2 weeks to monitor medication side effects. This assessment will be conducted by telephone by a trained trial nurse. Participants will be invited to come to hospital if there are indications of side effects on the telephone call. If the person cannot be reached by telephone, a health extension worker (community health worker) within the district will visit the person’s house to check. To facilitate this process, all participants will be linked to health extension workers, who will also be trained on talking to patients with mental illness and their families. To maximize adherence to follow-up assessments, we will also implement additional methods: (1) For participants to be recruited from Butajira, we will use our existing network of field workers and health extension workers to encourage follow-up. (2) For patients to be recruited from Addis Ababa: (a) we will only include persons with clear and traceable address. For example, we will exclude persons living in ‘NEW’ addresses. (b) We will use urban health extension workers. (3) For both sites, we will offer phone cards (Birr 15) to encourage contact by participants if they felt there was a clinical need to do so. We will also call a day before their scheduled follow-up appointment date to remind them of the appointment. Staff doing this will be trained to be courteous so that participants are treated respectfully and also are not harassed to attend.

### Assessors

PANSS, CGI, and GAF will be completed by either psychiatric nurses, masters-level trained psychiatry practitioners, or psychiatry residents or psychiatrists depending on their ability to conduct the assessments adequately after adequate training and assessment of inter-rater reliability. The OPCRIT will be administered by trained psychiatric residents (at least second year) or qualified psychiatrists after training. Physical assessment will be conducted either by psychiatric residents or general physicians. The OPCRIT will be initially validated against the SCAN. We will also assess the validity of the assessment by psychiatric nurses and residents, of all major instruments, against senior psychiatrists, as well as inter-rater reliability of assessments between the different professionals.

### Outcome

#### Primary outcome

Attained mean symptom severity at week 12 (based on total PANSS score) compared between the treatment arms; measured over three assessment time points: at entry (baseline), week 6, and end of intervention at 12 weeks.

#### Secondary outcomes

1. Change in severity of negative symptoms measured as difference in PANSS score between entry (baseline) assessment and end of intervention at 12 weeks.

2. Maintenance of change or improvement measured between weeks 12 and 16.

3. Change in CGI scores from baseline to week 12, measured as an absolute difference in scores.

4. Change in functioning measured using GAF from baseline to 12 weeks as a function of the mean difference in scores at the two time points.

5. Proportion attaining remission and time to attain remission defined using PANSS as per the definition of remission for schizophrenia [39].

6. Requirement of additional medication and dose adjustment; proportion requiring these additions and adjustments will be compared between the two arms.

7. Adverse effects of medications: proportion developing adverse effects as a function of severity will be compared in the two arms.

#### Tertiary outcome

Change in severity scores of the cognitive domain of the PANSS between baseline and week 12 will be compared between the treatment arms as a tertiary outcome.

### Data management and analysis

Data management will be handled by the Clinical trial unit at the Armauer Hanssen Research Institute (AHRI). This includes setting up data entry profile, handling interim analysis with the Data Safety and Monitoring Board (DSMB), and final analysis for the primary outcome. The primary analysis will be to compare the effect of treatment with minocycline compared with placebo in terms of attained mean symptom severity measured in PANSS score at week 12. Mixed effects model will be fitted to the PANSS score obtained at three time points (at baseline, 6 weeks, and 12 weeks) and trial treatments will be evaluated as a predictor of rate of change in PANSS score and attained PANSS score at week 12 adjusting for relevant baseline prognostic factors. These factors, identified as relevant for schizophrenia outcome, are duration of untreated psychosis, age of onset of illness, duration of illness, gender, and setting of recruitment (inpatient *versus* outpatient). Intention to treat analysis will be performed on primary outcome.

Secondary analysis will also be comparative.

–Analysis of measures at two time points (baseline and week 12 ) will compare absolute differences in symptom and functional scores (negative symptoms, GAF, CGI) between the treatment arms adjusting for relevant prognostic factors as proposed for the primary outcome.

–Profile of symptoms in the three domains of PANSS will be compared using repeated measures model: change and rate of change between the two treatment arms will be assessed with the mixed effects regression model.

–Time to remission will be computed using survival model. This will use the Cox Regression model adjusting for relevant prognostic factors as for the primary outcome.

–Sustainability of improvement or change will be measured as the proportion of those who have maintained their improvement (including those who have shown continued improvement) after 12 weeks compared between the two treatment arms. This comparison will rely on the PANSS, CGI, and GAF scores.

### Withdrawals and end of trial

Withdrawal from study may occur because of one of three reasons: refusal of participant to continue in study, occurrence of severe toxicity, and finally clear indication that the intervention is very effective and should be given to participants receiving placebo medication. In all cases, analysis will be by intention to treat. All protocol violations and major deviations will be recorded as they occur and will be included in reports of trial findings. The end of the trial will either be when the study period ends or by the recommendation of the DSMB if serious untoward events occur.

### Safety monitoring

Careful baseline assessment will be conducted so as to exclude potential participants who may experience serious adverse events, such as those with liver disease and renal disease. Adverse effects of medications will be monitored using biweekly (every two weeks) telephone calls by a trained clinical nurse. Participants will be encouraged to visit between the biweekly telephone assessments if they experience symptoms that are indicative of troublesome side effects. According to the safety profile of minocycline we expect very few side effects. In patients who received minocycline for schizophrenia for up to 12 months, no serious side effects were encountered [24].

We have established a DSMB, independent of the clinical trial, composed of a psychiatrist, pharmacologist, and statistician. All serious adverse events will be reported immediately to the DSMB and the Food, Medicine and Healthcare Administration and Control Authority of Ethiopia (EFMHACA) as well as the College of Health Sciences, Institutional Review Board (IRB), and National Research Ethics Review Committee (NERC). Minor adverse events will be reported as part of the regular reporting requirements. We will also have a trial monitor, who will be at least a general practitioner and would have training in Good Clinical Practice (GCP) and trial monitoring.

### Ethical approval

Ethical approval is obtained from the IRB of Addis Ababa University, College of Health Sciences (protocol number: 062/11/Psy), NERC (Reference number: 310/670/04), and the EFMHACA (reference number: 02/6/05/39). Appropriate consent procedures will be followed for the inclusion of participants in the study (Figure 1).

### Drug encapsulation and storage

Drug encapsulation procedure is provided separately. Both placebo and minocycline are formulated into opaque capsules. Minocycline capsules contain 100 mg of active ingredient and filler (Lactose NFP Powder) to ensure capsules are secure; the placebo will contain only filler in a total mass equal to that of the minocycline. Transportation can be done by regular (non-refrigerated) freight. Study drug will be stored at room temperature.

### Reporting

Results of the study will be reported to the IRB of the College of Health Sciences, the NERC, and EFMHACA in addition to being published in scientific literature.

## Trial status

Approval of the protocol has been obtained and recruitment of patients will begin within 2 months.

## Abbreviations

AHRI: Armauer Hanssen Research Institute; CGI: Clinical global impression; DSM-IV: Diagnostic and statistical manual of mental disorders; DSMB: Data safety and monitoring board; EFMHACA: Food, medicine and healthcare administration and control authority of ethiopia; GAF: Global assessment of functioning; GCP: Good clinical practice; IRB: Institutional review board; NERC: National research ethics review committee; OPCRIT: Operational citeria for research; PANSS: Positive and Negative syndrome scale for schizophrenia; SCAN: Schedules for clinical assessment in neuropsychiatry.

## Competing interests

The authors declare that they have no competing interests.

## Authors’ contributions

AF and TS conceived of the idea. AF led the development of the study plan and also led the drafting of the manuscript. MM helped drafting of the manuscript and coordinates the study. GM helped with designing the study methods and drafting of the statistical methods of the manuscript. MTL supported the development of the trial monitoring protocol. AA, ST, TG-E, TS, AA, JH, MTL, CB, DCH, and CH critically reviewed the manuscript. All authors approved final manuscript.
